# A novel molecular dynamics approach to evaluate the effect of phosphorylation on multimeric protein interface: the αB-Crystallin case study

**DOI:** 10.1186/s12859-016-0909-9

**Published:** 2016-03-02

**Authors:** Federica Chiappori, Luca Mattiazzi, Luciano Milanesi, Ivan Merelli

**Affiliations:** Institute for Biomedical Technologies, National Research Council (ITB-CNR), Segrate, MI 20090 Italy; Biotechnology and Biosciences Department, University of Milano-Bicocca, Milan, 20126 Italy

**Keywords:** Post-translational modification, Phosphorylation, Molecular dynamics, PCA, Clustering, αB-Crystallin, Chaperone, Small HSP

## Abstract

**Background:**

Phosphorylation is one of the most important post-translational modifications (PTM) employed by cells to regulate several cellular processes. Studying the effects of phosphorylations on protein structures allows to investigate the modulation mechanisms of several proteins including chaperones, like the small HSPs, which display different multimeric structures according to the phosphorylation of a few serine residues. In this context, the proposed study is aimed at finding a method to correlate different PTM patterns (in particular phosphorylations at the monomers interface of multimeric complexes) with the dynamic behaviour of the complex, using physicochemical parameters derived from molecular dynamics simulations in the timescale of nanoseconds.

**Results:**

We have developed a methodology relying on computing nine physicochemical parameters, derived from the analysis of short MD simulations, and combined with N identifiers that characterize the PTMs of the analysed protein. The nine general parameters were validated on three proteins, with known post-translational modified conformation and unmodified conformation. Then, we applied this approach to the case study of αB-Crystallin, a chaperone which multimeric state (up to 40 units) is supposed to be controlled by phosphorylation of Ser45 and Ser59. Phosphorylation of serines at the dimer interface induces the release of hexamers, the active state of αB-Crystallin. 30 ns of MD simulation were obtained for each possible combination of dimer phosphorylation state and average values of structural, dynamic, energetic and functional features were calculated on the equilibrated portion of the trajectories. Principal Component Analysis was applied to the parameters and the first five Principal Components, which summed up to 84 % of the total variance, were finally considered.

**Conclusions:**

The validation of this approach on multimeric proteins, which structures were known both modified and unmodified, allowed us to propose a new approach that can be used to predict the impact of PTM patterns in multi-modified proteins using data collected from short molecular dynamics simulations. Analysis on the αB-Crystallin case study clusters together all-P dimers with all-P hexamers and no-P dimer with no-P hexamer and results suggest a great influence of Ser59 phosphorylation on chain B.

## Background

Post-translational modifications (PTMs) regulate cell life. Several types of PTMs have been observed in proteins, alone or combined in order to regulate their activity and function. In particular, PTMs govern tertiary and quaternary protein structures [1]. One of the most extensively PTMs used by cells is phosphorylation. It regulates several cellular processes, among which are differentiation, growth, metabolism, apoptosis, cellular transport and signal transduction [2].

Phosphorylation consists in the esterification of a residue possessing a hydroxyl with phosphoric acid, a small and negatively charged group. This PTM can regulate a protein function by affecting its conformation and aggregation capabilities. Frequently, intrinsically disordered protein regions undergo to a structure rearrangement subsequently to a phosphorylation [3]. These structural rearrangements allow protein multimerization (small HSPs [4–6]), protein ordering (Microtubule Associated Protein Tau [7]), and protein-protein interactions (p47 [8]). Moreover, phosphorylation can affect the enzyme activity endorsing the ligand binding in the active site (Thymidylate synthase) [9, 10]. Frequently, the modification of multiple phosphorylation sites of a protein constitutes more than an on/off mechanism, since the level of phosphorylation can induce threshold related events. This mechanism is widely employed in eukaryotic regulatory proteins, like G protein-coupled [11] or tyrosine-kinase receptors [12], where the number of phosphorylated serine/threonine and tyrosine regulates the signal transduction. The availability of multiple phosphorylation sites in proteins provides a precise tool for dynamic regulation of the downstream processes. Different phosphorylation profiles of a single protein might be linked to different functions [13].

**Fig. 1:**
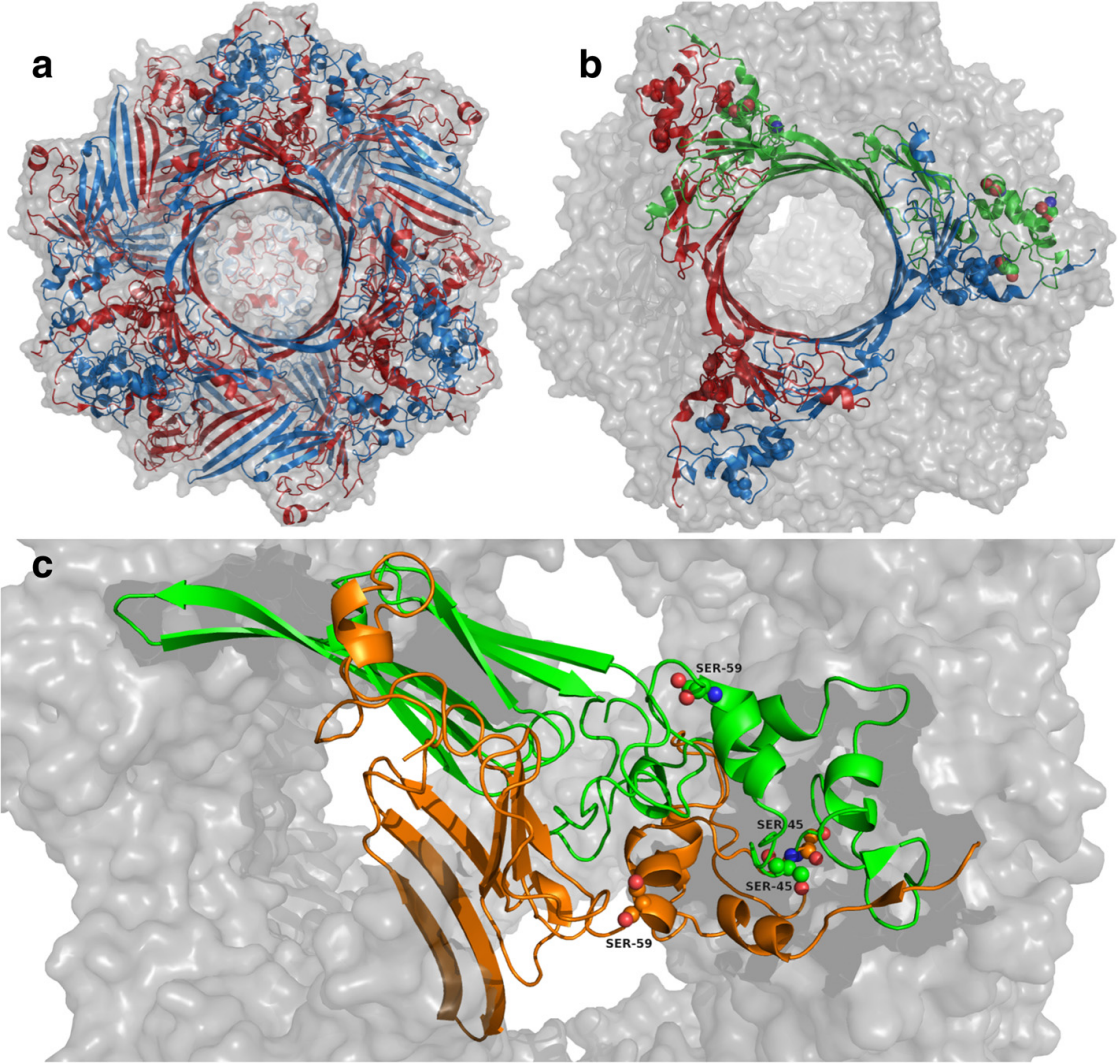
αB-Crystallin structure. **a** 24mer, in red the A chains and in blue the B chains. **b** hexamer, each dimer I (see Methods section for detail) is in a different colour; serine 19, 45 and 59 are in sphere. **c** dimer II, chain A is in green and chain B is in orange, Serine 45 and 59 are in sphere

A well-known class of proteins modulated by phosphorylation is the small Heat Shock Protein (HSP) family. Although for these proteins the role of phosphorylation is not completely understood, it is necessary to their chaperone function [2]. In particular, αB-Crystallin (HspB5) is a small HSP that is upregulated in several neuropathological diseases [14] as well as in different forms of cancer [15]. Both phosphorylated and unphosphorylated forms of αB-Crystallin have been found in eye lens, although the phosphorylated form increases with age [16]. This small HSP is also found in other tissues where it performs its role of chaperone, preventing intracellular aggregation of partially folded polypeptides [17]. Under cellular stress conditions, such as heat, oxidation, increase of intracellular calcium levels and ischemia, the level of the phosphorylated form increases [16]. αB-Crystallin is modulated by a trade-off between its different multimeric conformations. In particular, its simpler multimeric form is a homodimer, while more complex structures can be hollow globes composed from up to 20 homodimers [18] (Fig. 1a). The active units are smaller complexes, like the hexamer, which are torus-like structures composed by 3 dimers connected via an inter-dimeric interface [19] (Fig. 1b). The multimerization of this protein, and thus its function, is controlled by the phosphorylation of three serine residues, Ser19, Ser45 and Ser59. Phosphorylation of Ser19 has little effect on the chaperone activity [20] and no correlation with cytoprotection [21], while phosphorylation of Ser59 is important in controlling apoptosis and for the association of this small HSP with actin filaments [2, 22, 23]. Moreover, phosphorylation of Ser45 results in the disruption of the dimeric substructure induced by the steric hindrance of the two phosphate groups, which are too close to each other in the dimer [20]. Phosphorylation of Ser45 and Ser59 results also involved in αB-Crystallin localization at nuclear speckles [24]. Overall, in vitro and in vivo, only one or two serine residues result phosphorylated [25], for these reasons we included in this analysis only Ser45 and Ser59 as phosphorylatable serine (Fig. 1c). These residues are phosphorylated by the p44/42 mitogen-activated protein kinase and MAP kinase activated protein kinase 2, respectively [22]. Ser45 and Ser59 localize near the inter-dimeric interface, which supports the idea that phosphorylation influences the connection between dimers in higher structures. Aquilina et al. proposed that the disruption of dimeric interfaces is caused by serine phosphorylation, and this different conformation displays an increased affinity for the chaperone substrates [20]. Several works with engineered αB-Crystallin S19D/S45D/S59D or S19E/S45E/S59E can be found in literature, mimicking phosphorylation with aspartate or glutamate in tri-pseudo phosphorylated complexes [19, 24]. While changes in the multimeric state of αB-Crystallin were observed, only pure pseudo-phosphorylated monomers were used, without focusing on the behaviour of different αB-Crystallin in presence of pseudo-phosphorylated proteins [16].

Considering multi-PTMs involved in the multimerization level, the aim of this study is to develop a methodology to classify PTM patterns to predict their impact on the protein, by using data collected from short molecular dynamics (MD) simulations. In particular, MD simulations were extensively used to study conformational changes induced by Ser/Thr phosphorylations (tau peptide [7]; Na+/K+ - ATPase (NKA) [26], myelin basic protein (MBP) [27], and ADP ribosylation factor nucleotide site opener (ARNO) [28]). Nonetheless, simulations of large multimers in different phosphorylated conformations can take huge computational effort and impracticable analysis time. Therefore, we developed an approach to estimate the behaviour of structures by extracting physicochemical parameters from MD trajectories in the nanosecond timescale. Using this approach, structures resulting from different PTM patterns can be classified depending on their behaviour. According to our validation, this approach is reliable in discriminating the evolution of the system, caused by PTMs, relying on the variation of key indicators of the structure conformation and stability. Moreover, this approach has been applied to the different phosphorylation patterns of the αB-Crystallin in a 24meric state in order to identify the corresponding behaviour, starting from MD of dimeric structures.

## Results and discussion

### Parameter definition

In order to describe the effect of PTMs on protein structures, we focus on the structural and energetic differences between different PTM patterns. We define 9 physicochemical parameters (Table 1), which are combined with N case-specific identifiers related to the number of PTMs considered for the protein in analysis. To identify the peculiar characteristics of structures with different PTM states, we need the equilibrated conformations for each of these protein states. Therefore, all the considered structures must be simulated using MD in order to obtain an equilibrated conformation and a sufficient sampling for each of them. In detail, the 9 physicochemical parameters describe both the protein-protein interface and the protein complex from a structural and energetic point of view. Some of them are static parameters, which are calculated on representative conformations obtained from the equilibrated portion of MD simulations, while other represent the structural temporal evolution and therefore are evaluated on the whole trajectory.

**Table 1:** General parameters

Name	Type	Input for computation
Total SAS	Structural	Average value calculated on the eq trajectory
Hydrophobic SAS	Structural	Average value calculated on the eq trajectory
Buried SAS	Structural	Average value calculated on the eq trajectory
HB	Structural	Average value calculated on the eq trajectory
Gap_Index	Structural	Calculated on an average conformation
Volume	Structural	Calculated on an average conformation
E_LJ	Energetic	Average value calculated on the eq trajectory
AbsMin	Energetic	Calculated on FEL
#Min	Energetic	Calculated on FEL

The structural parameters evaluated on the equilibrated portion of the whole trajectories include: (1) Total SAS, (2) Hydrophobic SAS, (3) Buried SAS and (4) inter-chain hydrogen bonds number. SAS and the related hydrophobic SAS were evaluated according to Eisenhaber et al. [29], while Buried SAS was calculated as (Monomer 1 Total SAS + Monomer 2 Total SAS) - Complex Total SAS. Among all the hydrogen bonds established during the trajectory, only those involving residues of different chains were considered. (5) Gap_Index and (6) volume of the interface region are calculated on average conformations obtained from MD simulations. The first was described by Jones et al. [30], in order to better understand the protein-protein recognition mechanism, calculated on crystal structure of bound and unbound dimers, see Methods for details. Enclosed volume was calculated with the gap-sphere method described in [31]. Total SAS, Gap_Index and hydrogen bonds were previously described as parameters characterizing the interaction observed between proteins in the light of their biological function [30].

**Fig. 2:**
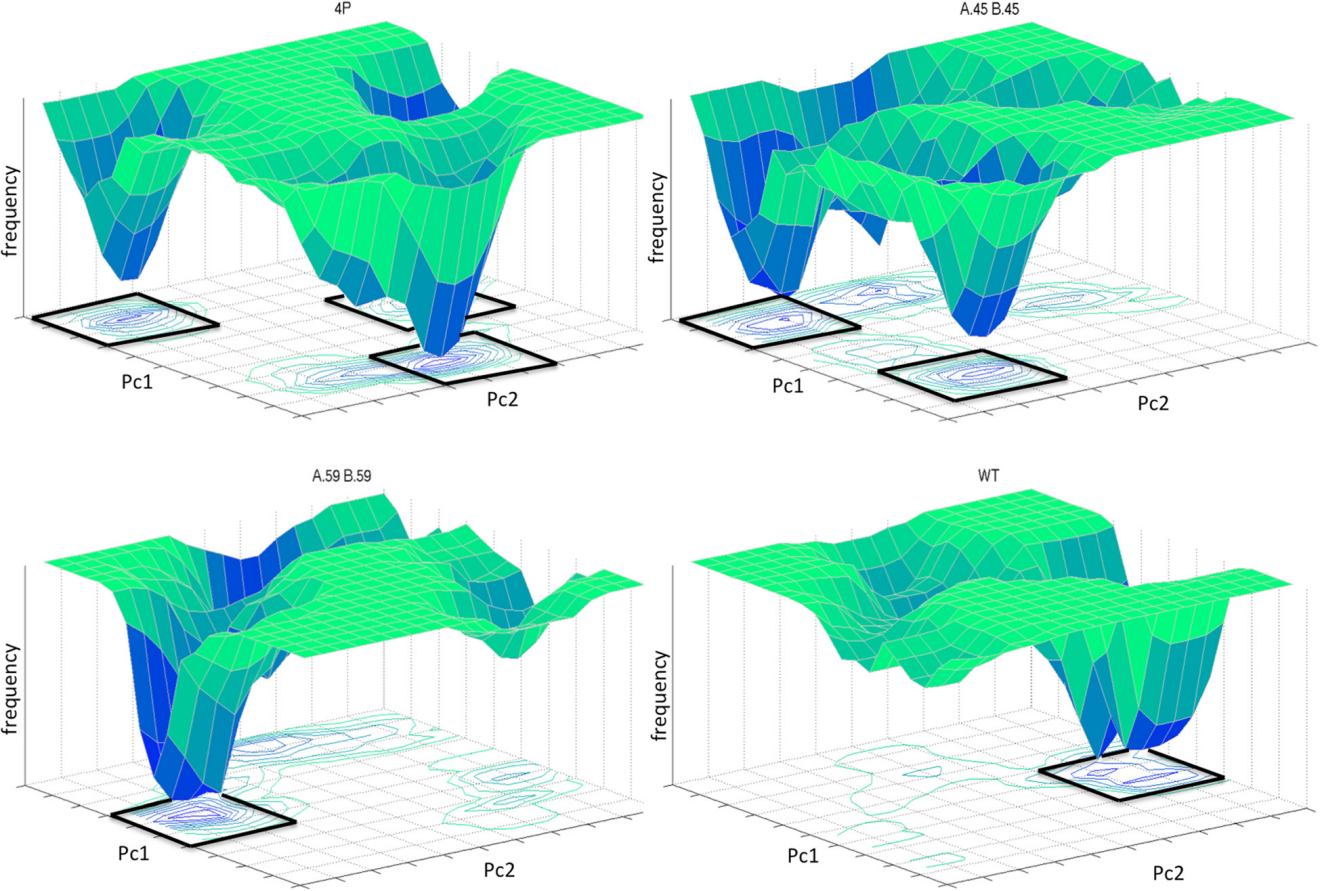
Examples of Free Energy Landscape. 3x3 boxes considered for the #Min value are in evidence

The energetic parameters were considered in order to take into account not only structural characteristics of the macromolecules, but also the differences in energy between bound and unbound protein complexes. These are average values from the analysis of the equilibrated portion of the whole MD simulation. In detail, the first value is the (7) inter-chain Lennard-Jones Energy, for which only the absolute value is taken into account. The other two values are obtained from the Free Energy Landscape (FEL), which representation is achieved projecting the trajectories on their first two principal components of motion [32, 33]. This representation returns the probability of finding the system in a particular state characterized by a combination of two reaction-coordinates values. The first two principal components of motion are the considered reaction-coordinates. From this analysis, the minimum frequency value (8), or better the probability of the most likely conformation, and the number of minima over a case dependent threshold of frequency (9) are collected. The latter is obtained applying a 20x20 points grid and summing the values included in a 3x3 box: boxes with values over the threshold are considered. Some examples are presented in Fig. 2. Parameters evaluated on the FEL are considered as markers of the protein stability. In detail, a stable conformation returns a limited number of large minima, while a larger number of minima characterize a meta-stable structure.

### Parameter validation

The 9 general parameters used by this approach were validated analysing three proteins for which both wild-type (wt) and post-translational conformations were experimentally available in the PDB. In particular, the validation of our approach has been performed on two X-ray structures of the Serine/threonine-protein kinase (TBK1) (PDB id: 4EUU/4EUT) [34], two NMR conformations of the CT10-Regulated Kinase isoform II (CRKII) (PDB id: 2DVJ/2EYZ) [35], and two crystal structures of the Glycogen Phosphorylase (GP) (PDB id: 8GPB/7GPB) [36].

TBK1 is a homodimer in its inactive form, and undergoes to a large structural rearrangement after phosphorylation of Ser172 in order to obtain the active conformation. Similarly to the αB-Crystallin the phosphorylation triggers a structural change that detaches regions of the 2 chains previously connected.

CRKII is a splicing isoform of the oncoprotein CRK. It is composed by three Src Homology (SH) domains, ordered according the SH2-SH3-SH3 pattern. Phosphorylation localizes in a linker region between the two SH3 domains, and induces the binding of the SH2 domain of CRKII itself, disrupting its biological activity.

GP exists as an inactive homotetramer and as an active homodimer. This protein was selected as validation benchmark given that the homodimer dimerizes when Ser14 is coupled with sulphate ions. Therefore, this is a negative example with respect to TBK1, CRKII, because the PTM induce the multimerization, resulting in a more compact structure without detachment of side chains. Our expectation is that wt GP will cluster together with the PTM conformations of TBK1 and CRKII (and finally with the phosphorylated αB-Crystallin), while the PTM conformation of GP will cluster with wt TBK1 and CRKII (and finally with wt αB-Crystallin). With this example we want to verify if our method is able to capture the global effect on the proteins conformation, connected with the structural rearrangement caused by PTM, instead of the local effects of the PTM (i.e. changes in the local charge, changes in the local shape).

**Fig. 3:**
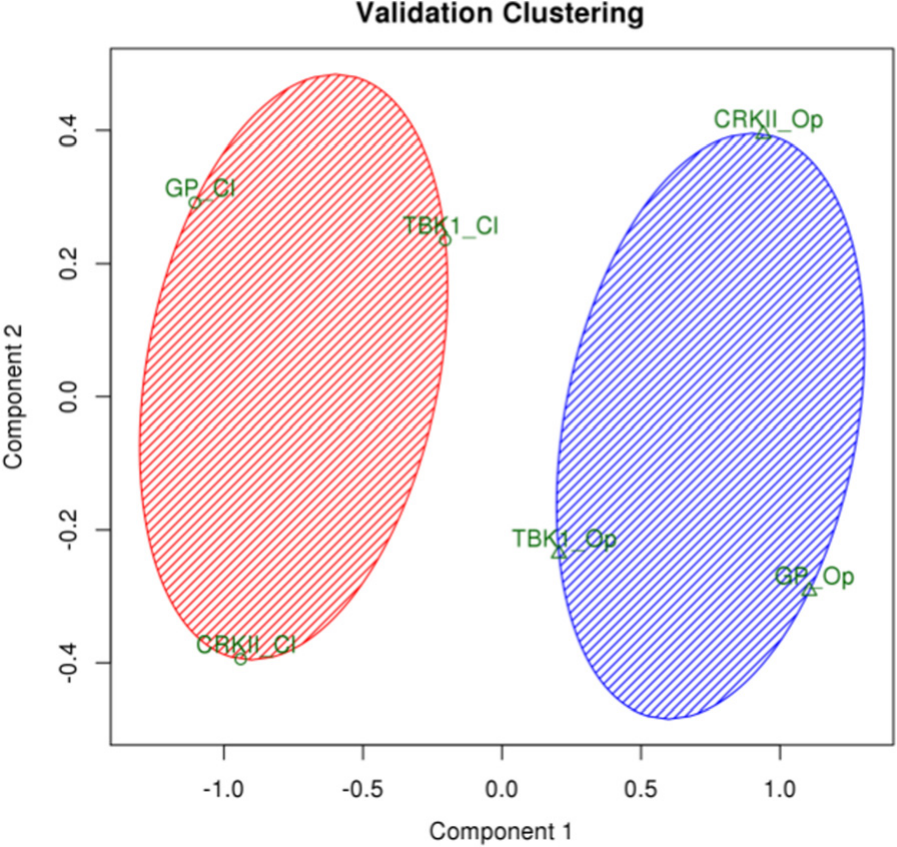
Clustering of the first 3PCs based on k-means algorithm. The three validation tests (GP, CRKII, and TBK1) both in PTM and wt conformations are represented

All six complexes, wt and post-translational modified conformations, were undergone to a short (10 ns) MD simulation, in order to collect the 9 general parameters. Data were normalized within each validation test by associating the wt protein with its modified version and dividing the value of each variable by the mean value of the two structures. The resulting normalized data was subjected to PCA in order to identify the most important parameters to characterize the protein behaviour. In particular, the structures were divided in 2 clusters using the k-means algorithm (the R implementation, which relies by default on the Hartigan–Wong algorithm) on the first 3 PCs (results are shown in Fig. 3) in order to discriminate the role of the different PTMs. The structures in which the presence of PTMs (or lack of PTMs in case of GP) induces the detachment of chains were clustered together (in Fig. 3 identified by the “Op” suffix). At the same way, structures in which the lack of PTMs (or presence of PTMs in case of GP) induces monomers aggregation were also clustered together (in Fig. 3 identified by the “Cl” suffix). This supports our claim that the 9 parameters are good predictors of the direction towards which the protein structure is shifting after a PTM.

### αB-Crystallin case study

The αB-Crystallin was chosen as case study, in order to clarify the role of phosphorylation on the multimerization state and on the achievement of the active conformation. Dimers and hexamers (Fig. 1), and the related phosphorylated forms, were obtained from the deposited 24-meric structure 2YGD, as described in the Methods section.

All complexes, 16 dimers and 2 hexamers, were simulated by MD for 30 ns, in order to obtain equilibrated conformations of unknown structures and a sufficient conformational sampling for the analysis. We defined four peculiar parameters according to the phosphorylation states of Ser45 and Ser59 of both chains A and B: in detail, value 1 was assigned to phospho-serine and 0 to the serine. Moreover, minima which frequency was 0.7 times the FEL absolute minimum were considered for the analysis. The parameters achieved for the different αB-Crystallin conformations underwent to a statistical analysis both to identify phosphorylation effects (parameters mainly correlated with the phosphorylatable residues) and to link phosphorylation patterns to measurable characteristics evaluated on protein structure.

We preliminary analysed the applicability of the approach to αB-Crystallin by mapping some of its conformers on the first two PC achieved during the parameter validations. In particular, all-phosphorylated and wt forms of the αB-Crystallin dimer II and hexamer were considered in the frame of the clusters achieved during validation. As expected, both the phosphorylated forms fall near the cluster centroid of the detached structures analysed in the validation examples, while wt conformers fall near the cluster centroid of the aggregated complexes. This preliminary example is a proof that the proposed approach is robust also for the analysis of our case study.

**Fig. 4:**
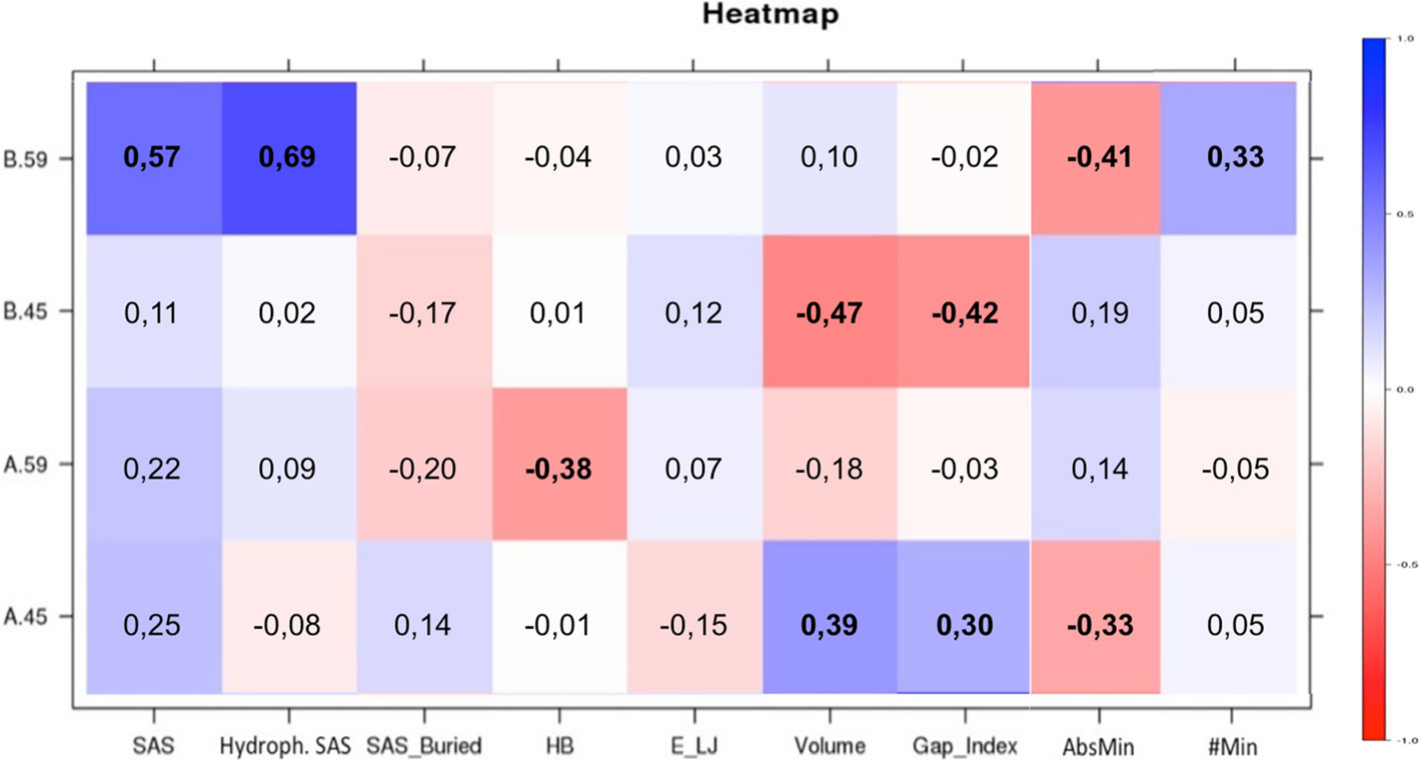
Correlation matrix. Representing the Pearson correlation coefficient between the phosphorylatable residues and the parameters employed in the analysis. Extreme colours represent high correlation between the two parameters, positive is in blue and negative is in red. Significant correlation is represented by values above |0.30| (in bold)

Now, considering the αB-Crystallin, PTMs, a Correlation Matrix between the general parameters and the specific phosphorylations of the protein was drawn, using the Pearson correlation coefficient (Fig. 4, correlation values are reported in each box), in order to identify the phosphorylation effects on macromolecular structures. Phosphorylation of residue A.45 and B.45 result correlated to Volume and Gap_Index, while only A.45 is negatively related to the minimum absolute value (AbsMin). Position 45 is at the dimer interface, its phosphorylation likely influence the distance between chains, represented by Gap_Index, and directly the inter-chains volume. Modification of A.59 residue was only negatively related to the average number of inter-chain hydrogen bonds, although this residue localize outward the dimer interface. Significant positive values were found between B.59 and Total SAS, Hydrophobic SAS and, to a lesser extent, the #Min, instead the minimum absolute value (AbsMin) is negatively correlated. Residue 59 localizes in the linker between ACD and CTD and chain B is in the bent conformation; since a large group is added in a cleft, this phosphorylation is likely to influence both Total SAS and Hydrophobic SAS.

### Principal Component Analysis

In order to study the key phosphorylations for αB-Crystallin activation, a PCA was applied considering all the 13 parameters, 9 general (Table 1) and 4 peculiar for the considered phosphorylations. The first 5 principal components were analysed, as they summed up to 84 % of the total variation and each of them had standard deviation > 1. Several methods for clustering the structures were tested on PCA data, in order to define which patterns behaved similarly, but the final choice was to use k-means because of the suitable possibility of integrating this algorithm with PCA (which in fact is known to be a relaxation of the k-means clustering [37]).

**Fig. 5:**
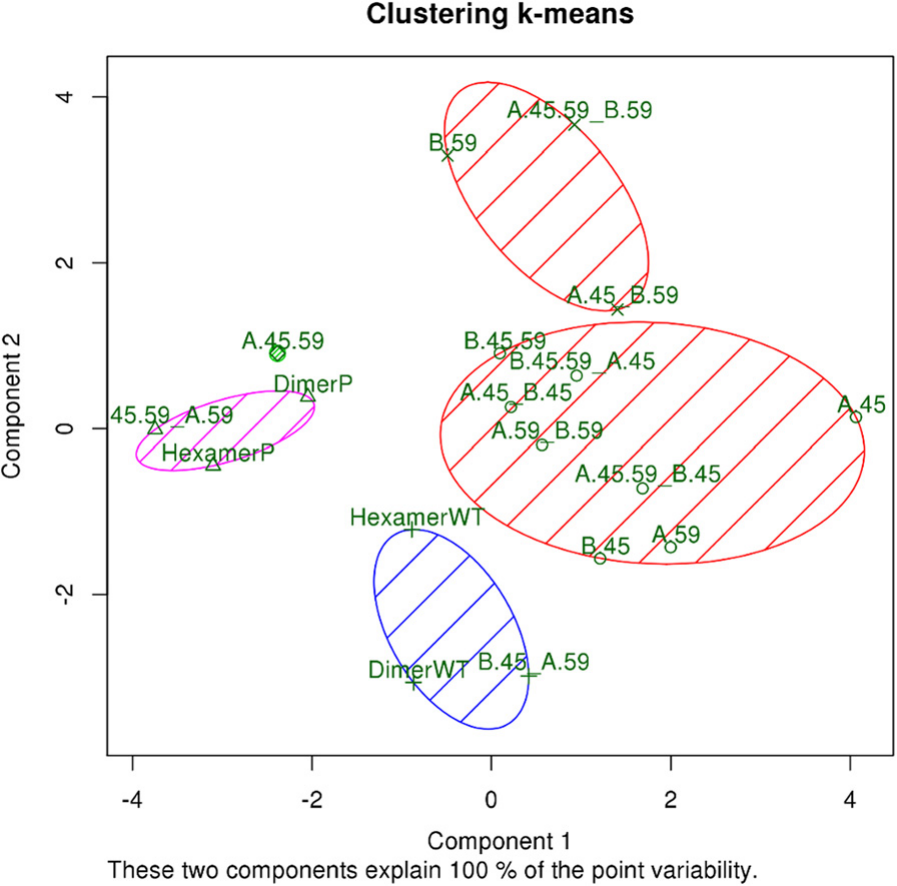
Clustering of PCA data based on k-means algorithm. The five clusters are evidenced with different colours

The k-means clustering algorithm, (selected after a comparison with other hierarchical clustering methods), returned the optimal solution with 5 clusters, resulting in a cluster containing all-phosphorylated dimer and hexamer and another cluster containing all the non-phosphorylated forms (the other clusters contain mixed phosphorylation patterns). Results of k-means algorithm using 5 clusters are displayed in Fig. 5. Both the dimer and the hexamer with all Ser45/Ser59 phosphorylated ended up in the same cluster with A.59-B.45.59. Non-P-dimer and non-P-hexamer also clustered together with A.59-B.45, suggesting a great influence of B.59 phosphorylation. A.45.59 stands alone in its cluster, B.59, A.45,59-B.59 and A.45-B.59 compose the fourth cluster, while all the remaining structures compose the fifth cluster. Other hierarchical clustering algorithms supported the same results, with phosphorylated structures more similar than the non-phosphorylated ones.

### Loading matrix interpretation

**Fig. 6:**
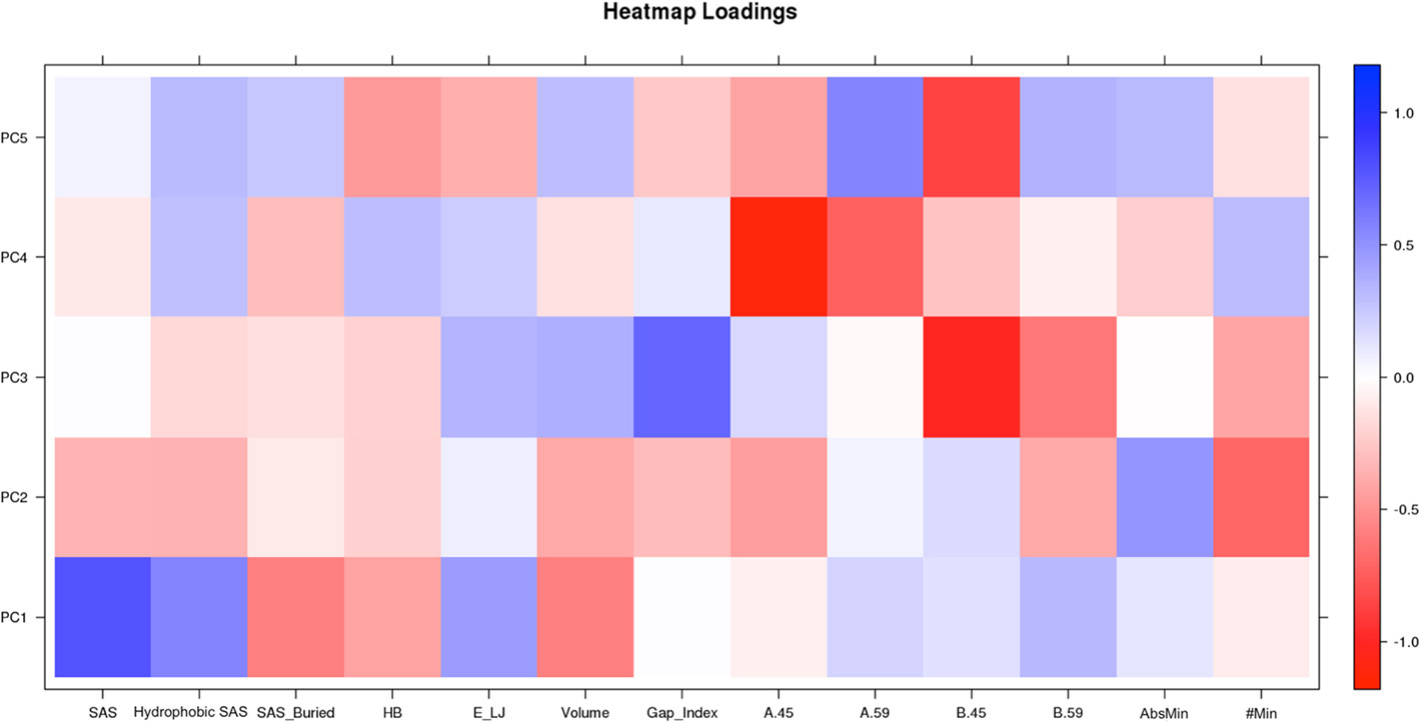
PCA factor loading matrix. Parameters are plotted against the first five PCs. Extreme colours represent mainly influencing variable to each PC

The variance reduction performed through PCA has been further investigated in order to identify which of the original parameters was more important in defining the higher Principal Components (PCs) and therefore was critical to discriminate between the behaviours caused by the different phosphorylation patterns. In particular, we analysed the loading matrix, as shown in Fig. 6, to verify how observed variables are expressed in terms of PC. Each row of the loading matrix describes the score (which values range between [-1;1]) of a variable for each PC, data are also collected in Table 2. Each PC is defined as the linear combination of the product of these values and their associated variables. Each loading can also be interpreted as a positive or negative correlation between an observed variable and a PC, while the most extreme values of loading indicate those variables that mostly define each PC. In detail, we can see how the Buried SAS positively influences the first component of the PCA and the Total SAS and Hydrophobic SAS variations, which account for almost half of the total variance described by PC1, negatively influence this. Considering PC2, we can see that it is positively influenced by #Min while there is a negative contribution of AbsMin: these two variables are also known to be anticorrelated. On the other hand, PC3 has a very important contribution from the Gap_Index and from B.45 phosphorylation state. At last, we can see that PC4 and PC5 are characterized by important contributions directly from the information concerning the residue phosphorylation (A.45 and A.59 positive for PC4, and B.45 - positive - and A.59 - negative - for PC5). The first 2 PCs show clear attributes from their most important variables: PC1 is mostly influenced by the geometrical structure of the protein, while PC2 groups the information about the stability.

**Table 2:** Loadings values

	PC1	PC2	PC3	PC4	PC5
Total SAS	0.405	−0.303	0.003	−0.104	0.042
Hydrophobic SAS	0.316	−0.347	−0.139	0.146	0.282
Buried SAS	−0.461	−0.047	−0.131	−0.163	0.247
HB	−0.378	−0.121	−0.199	0.229	−0.236
E_LJ	0.451	0.060	0.178	0.209	−0.203
Volume	−0.300	−0.351	0.202	−0.130	0.233
Gap_Index	0.001	−0.297	0.549	0.056	−0.216
A.45	−0.051	−0.226	0.157	−0.628	−0.411
A.59	0.171	0.030	−0.021	−0.565	0.289
B.45	0.130	0.128	−0.523	−0.247	−0.485
B.59	0.164	−0.350	−0.349	−0.062	0.275
AbsMin	0.065	0.479	−0.005	−0.115	0.274
#Min	−0.068	−0.364	−0.356	0.173	−0.132

### Experimental evidences supporting our results

Looking at the results of the αB-Crystallin case study, we can infer a great influence of Ser59 phosphorylation on chain B for the regulation of the protein multimerization. The key role of phosphorylation for the αB-Crystallin structure was widely demonstrated [16, 19, 20], as also the connection between phosphorylation level and chaperone activity [38]. In particular, phosphorylation of Ser59 has a role in ischemic stress, in the expression and regulation of cytoprotective proteins, like Bcl2 [39], and in the ubiquitin–proteasome system [40]. There are evidences that Ser59 phosphorylated αB-Crystallin accumulates in brains of patients with neurodegenerative disease, like Alexander and Alzheimer disease [41]. Moreover, inhibiting Ser59 phosphorylation leads to cell death [42]. These results support our claim that Ser59 is a key residue for determining the multimeric conformation of αB-Crystallin.

## Conclusions

PTMs, and in particular phosphorylations, have a great importance in the regulation of protein activity. Using molecular dynamics simulations to study their effects is a natural approach to the problem. Nonetheless, simulations of large multimers in different phosphorylated conformations can take huge computational effort (since very long simulations are necessary to reach an equilibrated conformation) and impracticable analysis time. Therefore, we developed an approach to estimate the behaviour of structures by extracting physicochemical parameters from MD trajectories in the nanosecond timescale. We validated our approach on two examples of structures known both in unmodified and post-translational modified states, achieving very good results in terms of predictions of the behaviour of the systems. In particular, the most interesting patterns which are able to describe the behaviour of the system from the very beginning of the simulation are the variation of the Total SAS, that is the geometrical structure of the protein, and the information about the stability (frequency and number of minima). Moreover, we applied our approach to different subunits of the 24meric structure of αB-Crystallin, showing that the parameters obtained from analysis of the asymmetric unit interface may predict the behaviour of the whole multimeric state.

## Methods

### Case study

The 24meric structure of αB-Crystallin (PDB id: 2YGD) has been obtained from PDB [25]. The monomer displays 2 conformations inside the oligomers, one bent and one straight. They assemble in *dimer I*, that can exist alone, and also in a different dimeric structure, named *dimer II*, that does not exist alone, but includes the inter-dimeric interface. *Dimer I* interface is composed by antiparallel β-strands of the α-Crystallin Domain (ACD), while in *dimer II* the interface is between the C-terminal Domain (CTD), where both the serines localize. We focused the analysis only on *dimer II*, as both phosphorylation sites are far from the interface in *dimer I*. We obtained the smaller units from the 24mer structure: *dimer II* and the hexamer. Using Chimera [43], we achieved all the 2^4^ = 16 combinations of the two possible phosphorylation residues (Ser45 and Ser59) for *dimer II*. Moreover, two hexamers were prepared for simulation: one with no phosphorylations and one with all the serines phosphorylated. Globally we modelled 18 structures, 16 dimers and 2 hexamers. In order to collect data for both the non-phosphorylated (non-P-) and the phosphorylated (P-) hexamer, we used the average of the values calculated for the structures composed by assembling *dimer II* monomers, since that interfaces can be both phosphorylated or not.

All structures have been solvated and neutralized with Na^+^ ions, then their free energy has been minimized using the Steepest Descent algorithm until the maximum force was smaller than 500 kJ(mol-1 nm-1). Then, a simulation of 30 ps in NVT environment was performed at 300 K, followed by 100 ps of simulation in NPT environment performed at 300 K and 1.0 bar.

### Molecular dynamics simulation and analysis

MD simulations of 30 ns at 300 K were obtained with Gromacs 4.5 [44], employing the amber99sbP force field, which includes an energy model for phospho-serines. All bonds were constrained using LINCS algorithm [45], and periodic boundary conditions were applied in all directions. Long-range electrostatic forces were treated using the PME method.

The representative conformation is the central structure of the first cluster obtained by clustering conformations sampled in the equilibrated portion of the trajectories, using the Gromacs tool, g_cluster on Ca atoms the gromos method [46] and applying a cut-off distance of 0.3 nm.

Equilibrated portion of the trajectories was evaluated based on RMSD plot. Representative conformations were evaluated using QMEAN [47] and Verify_3D [48] server. Ramachandran plots of the achieved structures were also analysed. Based on these evaluations, representative conformations quality is comparable to the 24-meric PDB structure (data not shown).

Energy evaluation, Hydrogen bonds analysis, Solvent Accessible Surface and RMSF were obtained using different tools from the Gromacs Suite, while data from the PCA of the trajectories was used for evaluating protein stability and metastable structures. Exploiting the Chimera plugin SurfNet, using 0.8 Å as grid interval and 5 Å as distance cut-off values, we obtained the volume of the interface region and its surface, and the ratio of volume on surface returns the Gap_Index (= Gap Volume (Å^3^)/ interface ASA (Å^2^)) [30].

R (http://www.r-project.org/) was used to obtain the Principal Component Analysis of the selected parameters, to cluster similar structures and to obtain the correlation matrix for the parameters and its heat map.

## Abbreviations

AbsMin: minimum absolute value
ACD: α-Crystallin Domain
CRKII: CT10-Regulated Kinase isoform II
CTD: C-terminal Domain
FEL: Free Energy Landscape
GP: Glycogen Phosphorylase
HSP: Heat Shock Proteins
MD: molecular dynamics
nonP-: non-phosphorylated
P-: phosphorylated
PCA: Principal Component Analysis
PTM: post-translational modifications
SAS: Solvent Accessible Surface
TBK1: Serine/threonine-protein kinase
wt: wild-type
